# Japanese Spotted Fever Complicated with Cholecystitis: A Case Report

**DOI:** 10.70352/scrj.cr.26-0048

**Published:** 2026-07-23

**Authors:** Junya Ishikawa, Satoru Ozawa, Norio Takemoto, Masatomo Kimura, Yusuke Miyazato, Masaki Nakamura

**Affiliations:** 1 Department of Surgery, Hashimoto Municipal Hospital, Hashimoto, Wakayama, Japan; 2 Department of Diagnostic Pathology, Hashimoto Municipal Hospital, Hashimoto, Wakayama, Japan; 3 Department of General Internal Medicine, Hashimoto Municipal Hospital, Hashimoto, Wakayama, Japan

**Keywords:** Japanese spotted fever, cholecystitis, rickettsiosis, vasculitis

## Abstract

**INTRODUCTION:**

Japanese spotted fever (JSF) is a tick-borne infection caused by *Rickettsia japonica*, classically presenting with the triad of fever, rash, and eschar, and characterized by systemic vasculitis resulting from endothelial injury. Concomitant cholecystitis associated with JSF is exceedingly rare.

**CASE PRESENTATION:**

We report the case of a 77-year-old man who presented with fever, was diagnosed with calculous cholecystitis at a referring hospital, and underwent laparoscopic cholecystectomy. Postoperatively, erythema developed on the extremities and trunk, along with an eschar on the right ankle. Polymerase chain reaction (PCR) testing subsequently detected *R. japonica*, confirming JSF. The ischemic injury secondary to vasculitis may have contributed to the development of cholecystitis.

**CONCLUSIONS:**

Even in calculous cholecystitis, JSF should be considered in endemic areas when rash or eschar is present, highlighting the importance of early PCR testing and the prompt administration of tetracyclines.

## Abbreviations

CRP
: C-reactive protein
DIC
: disseminated intravascular coagulation
JSF
: Japanese spotted fever
PCR
: polymerase chain reaction
SpO_2_
: peripheral oxygen saturation
WBC
: white blood cell count

## INTRODUCTION

JSF is a tick-borne infectious disease caused by *Rickettsia japonica*, characterized by infection of vascular endothelial cells.^1,2)^ Proliferation of the organism within endothelial cells induces systemic vasculitis, resulting in ischemic and inflammatory injury in multiple organs, including the skin, liver, kidneys, and lungs.^2,3)^ Clinically, JSF typically presents with fever, rash, and an inoculation eschar.^1,4)^ In severe cases, DIC and multiple organ failure may develop and can be fatal.^5,6)^

Reports of JSF complicated with cholecystitis are exceedingly rare,^1,2)^ and the pathogenesis and clinical implications remain unclear. Because the gallbladder has a relatively limited arterial supply, local ischemia driven by small-vessel vasculitis may contribute to its involvement.^2,3)^ Herein, we describe a rare case of JSF with concomitant cholecystitis and discuss potential pathogenic mechanisms based on the existing literature.

## CASE PRESENTATION

A 77-year-old man presented with abdominal pain. His medical history included hepatic dysfunction, chronic kidney disease, and hypertension. He had no history of alcohol consumption but smoked 10 cigarettes per day. The patient experienced persistent fever (approximately 38°C) for several days and visited a local clinic, where no obvious source of infection was identified and ceftriaxone was initiated. As his symptoms showed little improvement, he returned to the clinic a few days later; laboratory tests revealed worsening hepatic dysfunction. Imaging studies demonstrated gallbladder wall thickening as well as pleural and ascites effusions. Cholecystitis was suspected, and he was admitted. Because fever persisted with further hepatic deterioration, he was transferred to our hospital for further evaluation and management, including consideration of surgical intervention.

On admission to our hospital, the patient was alert and oriented. His height was 167 cm and weight was 74.5 kg. Vital signs were as follows: body temperature, 38.6°C; blood pressure, 153/78 mmHg; pulse rate, 122 beats/min (regular); respiratory rate, 30 breaths/min; and SpO_2_, 97% on room air. The abdomen was distended but soft, with tenderness extending from the right hypochondrium to the epigastrium without rebound tenderness or guarding. No rash or eschar was evident at that time. The results of laboratory examination were as follows: WBC, 14.7 × 10^3^/μL; CRP, 21.6 mg/dL; platelets, 6.2 × 10^4^/μL; aspartate aminotransferase, 253 U/L; alanine aminotransferase, 108 U/L; total bilirubin, 2.59 mg/dL; alkaline phosphatase, 417 U/L; gamma-glutamyl transpeptidase, 204 U/L (**Table 1**). Abdominal ultrasonography revealed gallbladder enlargement with wall thickening and multiple millimeter-sized gallstones. CT similarly demonstrated mild gallbladder wall thickening and gallstones, consistent with a diagnosis of acute calculous cholecystitis (**Fig. 1**). Given the diagnosis of acute calculous cholecystitis with systemic deterioration, laparoscopic cholecystectomy was performed. A small amount of straw-colored ascitic fluid was observed, and macroscopic inflammation of the gallbladder was mild. After induction of general anesthesia, multiple small patches of erythema were found on both thighs (**Fig. 2**). Histopathological examination of the resected gallbladder revealed mild cholecystitis. The subepithelial stroma showed mild inflammatory cell infiltration predominantly composed of plasma cells and lymphocytes, with focal neutrophilic infiltration within the mucosa. No definitive histopathological findings suggestive of vasculitis or ischemic change were identified. (**Fig. 3**). Postoperatively, the patient’s hemodynamic and respiratory status remained unstable, and he was admitted to the ICU for mechanical ventilation. On POD 1, there was no clinical improvement; thrombocytopenia and worsening hepatic dysfunction persisted. A systemic examination revealed progression of small erythematous lesions on the extremities and trunk, along with a 5-mm eschar on the right ankle. Tick-borne rickettsiosis was suspected; PCR and serologic testing were performed on samples from the eschar and blood, and minocycline therapy was initiated. As his respiratory condition failed to improve, the patient was transferred to a tertiary care hospital on the same day. Subsequent PCR testing detected *R. japonica*, establishing a definitive diagnosis of JSF.^7,8)^ Following transfer to that hospital, the patient gradually improved with continued treatment and was transferred to the general ward 9 days later. He was subsequently transferred back to our hospital for rehabilitation and was eventually discharged.

**Table 1:** Blood test results

Blood count	
WBC	14.7 × 10^3^/μL
RBC	4.49 × 10^6^/μL
Hb	13.5g/dL
Plt	6.2 × 10^4^/μL
Neutrophils	95.3%
Eosinophils	0%
Biochemistry	
CRP	21.6 mg/dL
Alb	2.8 g/dL
TP	5.7 g/dL
TBil	2.59 mg/dL
AST	253 U/L
ALT	108 U/L
γ-GTP	204 U/L
ALP	417 U/L
LDH	520 U/L
BUN	31.8 mg/dL
Cre	2.25 mg/dL
Na	137 mEq/L
K	4.4 mEq/L
HbA1c	6.7%

**Fig. 1:**
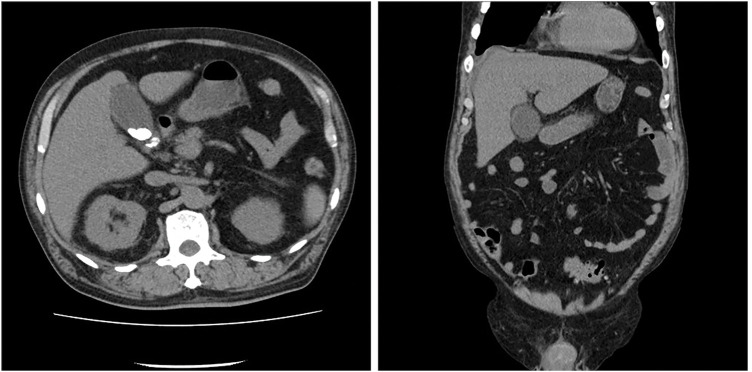
CT showing mild gallbladder wall thickening and a gallstone in the neck, indicative of calculous cholecystitis.

**Fig. 2:**
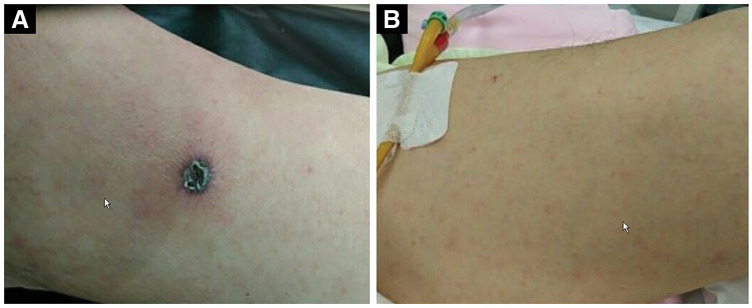
(**A**) An eschar indicating a tick bite on the right ankle. (**B**) Scattered small erythematous macules on both thighs.

**Fig. 3:**
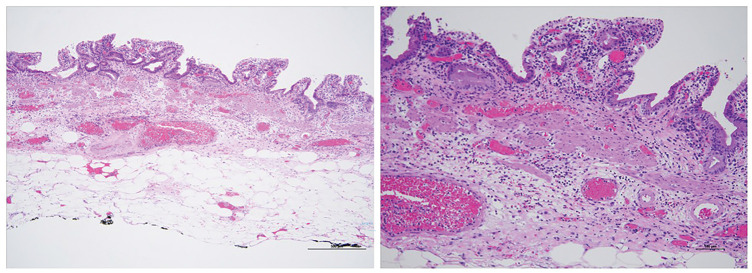
Histological findings showing mild cholecystitis. The subepithelial stroma exhibits mild inflammatory cell infiltration predominantly composed of plasma cells and lymphocytes, with focal neutrophilic infiltration within the mucosa.

In summary, the patient presented with persistent fever and liver dysfunction that failed to improve despite ceftriaxone therapy. Acute cholecystitis with systemic deterioration was suspected, and laparoscopic cholecystectomy was performed at our hospital. During induction of general anesthesia, erythematous lesions were noted on both thighs. Postoperatively, thrombocytopenia and liver dysfunction persisted, and a systemic examination on POD 1 revealed progression of the rash and the presence of an eschar on the right ankle. Given the suspicion of a tick-borne rickettsial infection, including JSF, minocycline therapy was promptly initiated, and PCR testing subsequently confirmed *R. japonica* infection. The patient’s condition gradually improved with continued treatment, allowing transfer to the general ward 9 days later.

## DISCUSSION

JSF is a tick-borne rickettsial disease caused by *R. japonica*, with many cases occurring in western regions of Japan.^4,9)^ Its characteristic clinical manifestations include acute fever, erythematous rash, and an eschar; however, an eschar is not consistently identified and may be overlooked during the initial evaluation. Previous domestic reports have indicated that fever and rash are present in nearly all patients, whereas an eschar has only been confirmed in 60%–70% of cases, potentially contributing to diagnostic delay. Laboratory abnormalities commonly include elevated hepatic transaminases, thrombocytopenia, and increased C-reactive protein levels, with liver dysfunction reported in approximately 70%–90% of patients. Because these clinical and laboratory findings are nonspecific, JSF may initially be difficult to distinguish from other conditions, such as acute cholecystitis, sepsis, or viral infections.^1,4)^ Although JSF generally responds well to tetracycline antibiotics, delayed initiation of an appropriate therapy has been associated with increased disease severity and mortality, underscoring the importance of early recognition and treatment.^1,6,9)^

Definitive diagnosis relies on serologic testing or PCR, including demonstration of a significant rise in antibody titers or detection of *R. japonica* DNA. PCR is particularly useful for early diagnosis around the time of rash onset; suitable specimens include blood, eschar, and skin-lesion biopsy samples, with eschar samples offering the highest sensitivity.^7,8)^

Rickettsial organisms infect vascular endothelial cells and proliferate within small vessels, resulting in endothelial cell swelling, perivascular fibrin deposition, and inflammatory cell infiltration. These vasculitic changes lead to impairment of the microcirculation and can subsequently cause multiorgan dysfunction involving the skin, liver, kidneys, and other organs.^2,3)^ In the present case, a characteristic rash and an eschar developed after laparoscopic cholecystectomy, and PCR testing confirmed *R. japonica*, establishing the diagnosis of JSF.^7)^

Cholecystitis associated with JSF is exceedingly rare, with only sporadic case reports found in the literature. Large clinical series contain little or no mention of cholecystitis, suggesting that it is an exceptionally uncommon complication.^4,9)^ The clinical features of fever, elevated liver enzymes, and inflammatory marker elevation closely mimic those of acute calculous cholecystitis, making preoperative diagnosis of JSF challenging. As observed in the present case, the appearance of skin rash or an eschar in the postoperative period serves as an important diagnostic clue of JSF.

The pathophysiology of cholecystitis in JSF differs from that of typical calculous or bacterial cholecystitis. In JSF, vasculitis-induced ischemic injury is considered the primary mechanism. The gallbladder is perfused by peripheral branches of the hepatic artery and is therefore vulnerable to microcirculatory impairment. Previous reports of severe JSF have described acute hepatic failure and systemic leukocytoclastic vasculitis, supporting the hypothesis that microvascular injury significantly contributes to hepatobiliary dysfunction.^5,6,10–12)^ Similar vascular-mediated mechanisms of cholecystitis have been documented in other rickettsial infections. In scrub typhus, several cases presenting with cholecystitis-like symptoms have been reported, and some patients showed resolution of fever and liver dysfunction only after initiation of doxycycline therapy, even when cholecystectomy had already been performed. These findings indicate that vasculitis-driven gallbladder inflammation may represent a shared pathophysiological mechanism among rickettsial diseases.^13–15)^

At our institution, 13 cases of JSF have been diagnosed since April 2024. Although several patients underwent evaluation for possible cholecystitis due to elevated hepatic enzymes, only the present case fulfilled diagnostic criteria for cholecystitis based on imaging findings and clinical course.

Although calculous cholecystitis could not be entirely excluded in the present case, the clinical course suggested that JSF may have contributed to the development of gallbladder inflammation. Histopathological examination did not demonstrate definitive evidence of vasculitis or ischemic injury, and therefore a causal relationship between JSF and cholecystitis could not be conclusively established. In endemic areas, even in patients with calculous cholecystitis, clinicians should maintain a high index of suspicion for JSF when patients present with cholecystitis-like symptoms, particularly when rash, eschar, or persistent postoperative fever is observed.

In summary, this case highlights the fact that cholecystitis in JSF may occur as a transient manifestation of vasculitic ischemic injury. Thus, early definitive diagnosis and prompt initiation of tetracycline therapy are crucial to prevent progression of severe organ dysfunction.^1,11,13–15)^

## CONCLUSIONS

In conclusion, JSF may rarely present with cholecystitis-like findings, and may have contributed to gallbladder inflammation through vasculitis-induced ischemic injury. In endemic areas, JSF should be considered even in patients with calculous cholecystitis when rash, eschar, thrombocytopenia, liver dysfunction, or unexplained systemic deterioration is present. Early PCR testing and prompt initiation of tetracycline therapy are essential to avoid diagnostic delay and prevent severe complications.

## DECLARATIONS

### Availability of data and materials

Data sharing is not applicable to this article as no datasets were generated or analyzed during the current study.
